# A pragmatic cluster randomised controlled trial to evaluate the safety, clinical effectiveness, cost effectiveness and satisfaction with point of care testing in a general practice setting – rationale, design and baseline characteristics

**DOI:** 10.1186/1745-6215-9-50

**Published:** 2008-08-06

**Authors:** Caroline Laurence, Angela Gialamas, Lisa Yelland, Tanya Bubner, Philip Ryan, Kristyn Willson, Briony Glastonbury, Janice Gill, Mark Shephard, Justin Beilby, members of the PoCT Trial Management Committee

**Affiliations:** 1 Discipline of General Practice, The University of Adelaide, Adelaide, South Australia, Australia; 2 Discipline of Public Health, The University of Adelaide, Adelaide, South Australia, Australia; 3 Save Sight Institute, University of Sydney, Sydney, New South Wales, Australia; 4 RCPA Quality Assurance Programs Pty Ltd, Adelaide, South Australia, Australia; 5 Community Point-of-Care Services, Flinders University Rural Clinical School, Flinders University, Adelaide, South Australia, Australia; 6 Faculty of Health Sciences, The University of Adelaide, Adelaide, South Australia, Australia

## Abstract

**Background:**

Point of care testing (PoCT) may be a useful adjunct in the management of chronic conditions in general practice (GP). The provision of pathology test results at the time of the consultation could lead to enhanced clinical management, better health outcomes, greater convenience and satisfaction for patients and general practitioners (GPs), and savings in costs and time. It could also result in inappropriate testing, increased consultations and poor health outcomes resulting from inaccurate results. Currently there are very few randomised controlled trials (RCTs) in GP that have investigated these aspects of PoCT.

**Design/Methods:**

The Point of Care Testing in General Practice Trial (PoCT Trial) was an Australian Government funded multi-centre, cluster randomised controlled trial to determine the safety, clinical effectiveness, cost effectiveness and satisfaction of PoCT in a GP setting.

The PoCT Trial covered an 18 month period with the intervention consisting of the use of PoCT for seven tests used in the management of patients with diabetes, hyperlipidaemia and patients on anticoagulant therapy. The primary outcome measure was the proportion of patients within target range, a measure of therapeutic control. In addition, the PoCT Trial investigated the safety of PoCT, impact of PoCT on patient compliance to medication, stakeholder satisfaction, cost effectiveness of PoCT versus laboratory testing, and influence of geographic location.

**Discussion:**

The paper provides an overview of the Trial Design, the rationale for the research methodology chosen and how the Trial was implemented in a GP environment. The evaluation protocol and data collection processes took into account the large number of patients, the broad range of practice types distributed over a large geographic area, and the inclusion of pathology test results from multiple pathology laboratories.

The evaluation protocol developed reflects the complexity of the Trial setting, the Trial Design and the approach taken within the funding provided. The PoCT Trial is regarded as a pragmatic RCT, evaluating the effectiveness of implementing PoCT in GP and every effort was made to ensure that, in these circumstances, internal and external validity was maintained.

**Trial Registration:**

12612605000272695

## Background

Point of care testing (PoCT) has been used for many years and is increasingly being utilised in the Australian general practice (GP) setting. PoCT is defined as any test that is performed at the time at which the test result enables a clinical decision to be made and an action taken that leads to an improved health outcome [1]. PoCT has the potential to provide better monitoring of chronic conditions, improved therapeutic control, more rational prescribing, better clinical decisions within the consultation timeframe, greater patient compliance with pathology requests, and fewer visits to the doctor [2-4].

The literature suggests that there is a lack of evidence for several of these benefits, particularly those relating to clinical outcomes. Reduction in referrals, and earlier and more rationalised treatment has been reported in a study involving PoCT [5] but changes in prescribing patterns have not occurred. Some evidence is available on the role of PoCT in improving glycaemic control [2,6], cholesterol and lipid levels [7], and oral anticoagulant control [8], although not for microalbuminuria.

A primary concern relating to PoCT is quality management. For PoCT to be introduced into the GP environment, it is important that it is proven to be accurate and reliable [9]. This requires those practices undertaking such testing to meet both internal quality control (QC) and external quality assurance (QA) standards. Hobbs [10] suggests a model for PoCT in primary care that incorporates laboratory training for GP staff with external QA from a central laboratory. A necessary part of quality management is the adequate training of staff operating the PoCT devices and this includes the requirement for an understanding of QC and QA processes [11,12].

PoCT in GP will only be effective if the results obtained from the testing devices are comparable with laboratory results [1,13]. A number of studies have shown that PoCT using a variety of portable monitoring devices can produce test results similar to laboratory results for a number of specific tests including HbA1c [2,14], anticoagulation monitoring [15-19], microalbuminuria and cholesterol [20]. Variability between laboratories and primary care sites, however, demonstrates the need for participation in QA programs [1].

While a large number of studies have been undertaken on the use of PoCT in the primary care setting, few studies have undertaken an economic analysis of PoCT [5]. Hobbs et al's [3] systematic review of PoCT in primary care could not draw any conclusions regarding cost effectiveness of PoCT because of insufficient research data. Some studies indicate that PoCT is more expensive when compared to laboratory testing [2,21], but this may be offset by long term societal gains such as prolonged life or reduced hospital stays [5]. It should, however, be noted that the cost effectiveness of PoCT appears likely to vary according to the disease group and the test in question [2].

The attitudes of key stakeholders and their satisfaction with PoCT form an important part of the assessment of introducing PoCT into GP. These stakeholders include patients, GPs, practice staff and pathology laboratories. PoCT may lead to greater convenience for GPs and patients but result in greater costs and require organisational changes that may reduce stakeholder satisfaction. There is conflicting research in this area. Hilton et al's [22] study on general practitioner and practice nurse attitudes to PoCT concluded that GPs did not find PoCT a useful addition to their practice, while nurses reported that pressure on their time was a limitation for PoCT. Grieve et al. [2] found no difference in patient satisfaction between diabetes clinics using PoCT or usual laboratory testing, however, patients did record a higher level of satisfaction with test information if they had PoCT rather than conventional testing. An Australian study investigating the attitudes of patients and GPs to PoCT found that GPs and patients supported PoCT because of its convenience, quality, role in patient care and efficiency. However, registration costs and QA fees were cited as areas of dissatisfaction by GPs [23].

While a number of studies have been undertaken on the cost effectiveness, clinical effectiveness, safety or satisfaction with PoCT, there have been no randomised controlled trials (RCTs) that evaluate all these outcomes in the GP setting. The PoCT Trial was implemented to address this gap.

## Methods

### Overview of design

The PoCT Trial is a cluster randomised controlled trial to evaluate the intervention of PoCT on the management of patients with either diabetes, hyperlipidaemia or on anticoagulant therapy. A clustered design was chosen to avoid treatment group contamination and for administrative convenience. The Trial commenced in September 2005 and continued for 18 months across 58 general practices based in urban, rural and remote locations across three states in Australia.

The Trial Design was developed in partnership with the PoCT Steering Group, a working group of the Quality Use of Pathology Committee PoCT Implementation Subcommittee of the Australian Government [24]. The original Trial Design was then modified by the Trial Evaluators (CL, AG, PR, LY, KW) and the evaluation protocol and statistical analysis plan developed for the adapted Trial Design.

The PoCT Trial involved collaboration between three organisations who administered different aspects of the Trial. Trial Management and Evaluation was undertaken by the Disciplines of General Practice and Public Health at the University of Adelaide. The provision of devices, device operator training and QC was undertaken by the Community Point-of-Care Services Unit, Flinders University Rural Clinical School (Device Group) and the external QA program was provided by the RCPA Quality Assurance Program Pty Ltd (QAP).

The primary research question of the Trial was:

Should PoCT in GP be implemented by the Australian Government?

The Trial evaluated seven key questions:

1. Is it safe to perform PoCT in a GP setting?

2. Is the clinical effectiveness of PoCT the same or better than the same tests using pathology laboratory testing?

3. Is it the same or more cost effective to perform PoCT compared with pathology laboratory testing?

4. Are patients and other stakeholders more satisfied with PoCT than with pathology laboratory testing?

5. Are there differences between urban, rural and remote geographic regions in the treatment effects being measured?

6. Would the regulatory environment used for the Trial meet the needs of all the stakeholders if PoCT were to be made more generally available?

7. What would the appropriate MBS fees be for the PoC tests selected in the Trial?

This paper focuses on the evaluation protocol and methods developed to investigate the first five research questions.

A number of hypotheses were developed in order to answer each of these questions and are shown in Table 1.

**Table 1:** Key research questions and associated hypotheses

**Area of key** ** research question**	**Hypotheses developed**	**Level of analysis**
Safety	All designated staff in the PoCT practices meet the required competency level to perform PoCT	Practice staff level
	All PoCT practices obtain QC results within the acceptable performance range	Practice level
	In terms of accuracy, all PoCT practice results meet the required QA performance levels for the pathology laboratories	Practice level
	In terms of precision, PoCT practice results meet the required QA performance levels for the pathology laboratories	Practice level
	Results obtained from PoCT devices for each patient closely agree with results obtained for the same patient from pathology laboratory testing	Test level
	All intervention practices meet the standards for PoCT in GP and obtain accreditation	Practice level
	The number of serious adverse events reported in PoCT patients per person-year is the same as or fewer than the number of serious adverse events reported in control patients per person-year	Patient level
	The proportion of PoCT patients who experience one or more serious adverse events is the same as or less than the proportion of control patients who experience one or more serious adverse events	Patient level

Clinical effectiveness	The proportion of PoCT patients who have pathology results within the target range is the same as or greater than the proportion of control patients who have pathology results within the target range	Patient level
	The proportion of total tests within the target range in PoCT practices is the same as or greater than the proportion of total tests within the target range in control practices	Test (within patient) level
	The number of general practitioner visits for PoCT patients per person-year is different than the number of general practitioner visits for control patients per person-year	Patient level
	PoCT patients report the same or greater improvement in compliance with disease management as directed by medical staff as control patients	Time of questionnaire administration (within patient) level

Satisfaction	The average change in attitudes in GPs from PoCT practices is different to the average change in attitudes in GPs from control practices	Time of questionnaire administration (within GP) level
	The average change in attitudes in patients from PoCT practices is different to the average change in attitudes in patients from control practices	Time of questionnaire administration (within patient) level
	Device operators report a change in average attitudes	Time of questionnaire administration (within device operator) level
	Pathology providers report a change in average attitudes	Time of questionnaire administration (within pathology provider) level
	The average level of satisfaction with regard to PoCT assisting with disease management in intervention GPs is different to the average level of satisfaction with disease management in control GPs	GP level
	The average level of satisfaction with regard to work flow in intervention GPs is different to the average level of satisfaction with regard to work flow in control GPs	GP level
	The average level of satisfaction with testing in intervention GPs is different to the average level of satisfaction with testing in control GPs	GP level
	The average level of satisfaction with regard to the collection process in intervention patients is different to the average level of satisfaction with regard to the collection process in control patients	Patient level
	The average level of confidence in the process in intervention patients is different to the average level of confidence in the process in control patients	Patient level
	The average level of confidence in the results in intervention patients is different to the average level of confidence in the results in control patients	Patient level
	The average level of satisfaction with regard to transport in intervention patients is different to the average level of satisfaction with regard to transport in control patients	Patient level
	The average level of satisfaction with regard to loss of work time in intervention patients is different to the average level of satisfaction with regard to loss of work time in control patients	Patient level
	The average level of satisfaction with regard to disease compliance in intervention patients is different to the average level of satisfaction with regard to disease compliance	Patient level
	Device operators are satisfied with PoCT	Device Operator level
	Pathology providers are satisfied with PoCT	Pathology Provider level

Cost effectiveness	The value of the resources used in PoCT is different from that of those used in pathology laboratory testing	Patient level

The PoCT Trial had two phases, Phase I lasting six months and Phase II lasting twelve months. In Phase I, patients in practices in the intervention group had pathology testing performed both by the pathology laboratory in the usual manner and by PoCT in the practice. The control group undertook testing by the pathology laboratory.

In Phase II, intervention group patients were tested using only PoCT at the practice, although pathology laboratory testing could be performed at any time at the request of the general practitioner, while control group patients continued to be tested by the pathology laboratory as usual.

### Study setting

The Australian Medicare Program aims to provide equitable access to medical and hospital services for all Australian residents. The Department of Health and Ageing (the Department) has policy responsibility for Medicare, with Medicare Australia being responsible for Medicare administration and the payment of Medicare benefits, which are detailed in the Medicare Benefits Schedule (MBS). The Medical Services Advisory Committee (MSAC) advises the Department on the strength of evidence relating to the safety, clinical effectiveness and cost effectiveness of new and emerging medical services and technologies, and under what circumstances listing on the MBS should be supported.

To enable the payment of Medicare benefits for pathology services, pathology testing must be undertaken by a pathology laboratory that is accredited to provide a particular service. Pathology collection centres must be approved to enable the payment of Medicare benefits for services performed on specimens collected. Patients can either attend an approved collection centre or can have their blood, urine or other samples taken by or on behalf of the general practitioner referring them for the service. The specimen is then delivered to the pathology laboratory for testing. For patients in rural and remote locations, transportation time is critical and turnaround time for results can be longer than in urban settings, with a possible impact on patient management.

Currently in Australia only a small number of point of care tests, such as pregnancy tests, are funded through the MBS for GP. To claim a broader range of pathology tests a practice must be a Category M (GP) Accredited Pathology Laboratory. This requires that they participate, at a cost, in the inspection and accreditation process implemented by the National Association of Testing Authorities. It has been suggested that the costs associated with accreditation and annual registration currently make it prohibitive for GP to participate, resulting in very few such practices existing in Australia [23]. The outcomes of the PoCT Trial will be used by the Department and its relevant advisory bodies (including MSAC) to determine whether an expanded range of PoCT for GP should be recommended for inclusion in the MBS.

### Participants

The Trial aimed to recruit 60 practices across three geographic locations (urban, rural and remote) in South Australia, New South Wales and Victoria. Node support officers (NSOs) were employed across the three geographic regions with the aim of recruiting 20 practices each. Divisions of GP were engaged to assist in the recruitment process. Once a general practitioner or practice had expressed an interest in being involved in the Trial, further information was sought in the form of a practice checklist. Those who met the eligibility criteria and signed on to participate were included in the randomisation.

All practices were required to recruit a minimum of 30 patients on anticoagulant therapy, 35 with diabetes and 50 with hyperlipidaemia. Patients were initially recruited in the first three months of the Trial. Patients needed to have established and stabilised diabetes, and/or hyperlipidaemia, and/or be taking anticoagulant medicine such as warfarin. The inclusion and exclusion criteria for practices and patients are provided in Table 2. Practices were provided with assistance to undertake database searches to identify eligible patients and a software program to randomly select eligible patients to be invited to participate in the Trial. These patients were sent, by the practice, a letter of invitation, an information sheet and a consent form. Once patients had consented, they were provided with an identification card with their ID number and those in the intervention group began PoCT.

**Table 2:** Inclusion and Exclusion criteria for practices and patients in the PoCT Trial

**Participant** ** Group**	**Inclusion criteria**		Exclusion criteria
Practices	Accredited through RACGP Standards for GP Minimum patient load from all disease groups: 20 tests per month INR 10 test per month Lipids 5 tests per month HbA1c Suitable facilities for PoCT – premises, staff and medical record system		Involved in other primary care pathology trial

Patients	Diabetes	Fasting plasma glucose ≥ 7.0 mmol/L or 2 hour post glucose load ≥ 11.1 mmol/L	< 18 years Condition not stabilised Unable to understand written instructions in English
	Hyperlipideamia	Eligible for PBS lipid lowering drugs	Significant cognitive impairment
	Anticoagulant therapy	Prescribed Warfarin INR test result within the therapeutic range for at least one month (ie stabilised)	Poor insight into their disease process or physical disabilities

All GPs from each practice involved in the Trial were invited to participate. Those interested in participating were required to sign a consent form, to sign a letter of agreement which outlined the terms and conditions of their participation in the Trial and were asked to provide proof of their current Medical Malpractice Insurance.

Participating practices were required to provide contact details for all associated pathology providers. The Trial Management team encouraged these pathology providers to participate in the Trial and developed appropriate links for data transfer where possible to reduce the burden on practices.

Practices randomised to the intervention group were required to nominate at least one staff member, preferably a practice nurse, to undertake training in use of the PoCT devices and the Trial protocol.

### Randomisation and allocation

Practices were randomly allocated to the intervention or control arm in the ratio 1:1. Randomisation was stratified by geographic area (urban, rural and remote) and used randomly permuted blocks of size 2, 4 and 6. The random allocation sequence was generated using ralloc.ado version 3.2.5 in Stata 9.0.

Practice allocation to treatment group was performed by central randomisation by phone/email after consent was obtained. The recruiters did not know the random allocation sequence. Once randomisation had been undertaken, all the patients recruited for the practice were then deemed intervention or control depending on which arm of the Trial each practice was allocated.

Due to the type of intervention, neither participants nor project staff were blinded to the treatment allocation.

### Intervention

Patients recruited in the intervention group had their pathology testing for either HbA1c, microalbumin, lipids (total cholesterol, triglyceride and high density lipoprotein cholesterol (HDL-C)) or INR using PoCT devices by their practice for 18 months. Patients in the control practices had these same tests undertaken using their usual care – the pathology laboratory.

The practices in the intervention group were provided with three PoCT devices – DCA 2000, CoaguChek S and Cholestech LDX. The selection of devices was based on non-analytical and analytical criteria prior to the commencement of the PoCT Trial. Group training sessions were held over two days for the practice staff who would use the devices (known as device operators) and follow-up sessions were provided 12 months later. Training incorporated an introduction to PoCT, quality management, accuracy and precision of results and training in the use of each device. At the end of their training, their competency was assessed by the Device Group.

#### Quality Management

The practices were required to perform internal QC and QA testing designed for the PoCT Trial practices. The practices also participated in an accreditation process. The accreditation process and training was based on the Interim Standards for Point of Care Testing in General Practice: Incorporating the Trial Guidelines developed by the Australian Government [25].

### Data collection and outcome measures

Data were collected at various points throughout the PoCT Trial to answer the five key research questions (see Additional file 1). In determining the data collection tools and outcome measures, it was necessary to ensure that these were applicable across the three different clinical conditions and could accommodate patients with more than one of the three conditions. Thus, validated tools or measures appropriate to only one condition could not be used. The Evaluation Team developed unique data collection tools and also utilised validated tools where applicable, and these are described below.

#### Safety

To determine the safety of PoCT, a number of measures were identified. These included: performance in quality management; performance testing; compliance with standards for PoCT; and serious adverse events (SAEs) reporting.

Internal QC for the intervention practices was assessed using QC materials comprising two levels of HbA1c, microalbumin and lipids and one level for INR. Device operators forwarded their results to the Device Group for analysis. This was initially undertaken fortnightly and after three months undertaken monthly for the remainder of the Trial. Practices received a feedback report every three months reporting their precision expressed as a coefficient of variation.

The QAP provided an external assessment of the PoCT device performance and a comparison with all practices in the Trial. Intervention practices were provided with an external QA kit for each test every six months during the Trial. The kit contained samples which needed to be tested every fortnight by the device operators. Practices forwarded results by mail or via the QAP website to QAP Pty Ltd and acceptable limits of good performance were determined. At the end of each testing cycle, practices were provided with a summary of their performance (precision and accuracy) over the previous six months and a comparison with other participating sites.

The number and types of SAEs were recorded throughout the length of the PoCT Trial. Events occurring up to one month after completion of Phase II were also recorded for patients on anticoagulant therapy. The number of SAEs reported in patients per person-year was calculated, as well as the proportion of patients experiencing one or more SAEs.

#### Clinical effectiveness

To determine the clinical effectiveness of PoCT compared with pathology laboratory testing, the PoCT Trial focused on therapeutic control and impact on patient care.

To measure therapeutic control, two outcomes were considered: firstly, the proportion of patients within target range (prevalence) and secondly the proportion of tests within target range for each type of test. The former was the primary outcome measure for the Trial. The target ranges used for the seven tests were based on clinical guidelines and are defined in Table 3. For intervention practices, patient test results were recorded on a specifically designed request/result form, with copies forwarded to the Trial Manager every month. For control practices, test results were collected from the pathology laboratory through either hard copy or weekly electronic downloads of results for those patients identified as participating in the Trial. This was achieved with a PoCT Trial identification sticker adhered to the pathology laboratory test request form.

**Table 3:** Target ranges by condition and test

**Condition**	**Test**	**Target range**
Diabetes [39,40]	HbA1c	< = 7%
Microalbuminuria [40]	ACR	< 3.6 female
		< 2.6 male
	Urine albumin	> 20 μg/min

Hyperlipidaemia [42]	Total cholesterol	< 4.0 mmol/L
	Triglycerides	< 2.0 mmol
	HDL-C	> 1.0 mmol/L

INR [41]	Atrial fibrillation and other conditions	2.0–3.0
	Prosthetic heart valve	2.5–3.5

To measure the impact of PoCT on patient care, a number of outcome measures were determined. These included: number of general practitioner visits per person-year; patient compliance with disease management; pharmaceutical prescribing; and process of care actions undertaken by the general practitioner following a pathology test.

#### General practitioner visits per person-year

Pathology results provided by PoCT at the time of a consultation provide GPs with an opportunity to discuss the test results with their patients immediately and implement any changes to improve the management of their condition. Measuring the number of general practitioner visits per person-year for patients in the PoCT Trial will determine if PoCT leads to more or fewer visits. Some research indicates that PoCT results in increased testing [26], while others have found no significant difference [27,28].

#### Medication compliance

It has been widely reported in the literature that non-compliance to medication is substantial with an estimated 30–40% of patients failing to take medications as prescribed [29]. It is well known that low compliance to medication compromises the effectiveness of treatment at substantial costs to the patient (to the potential detriment of health), to the health professional (treating morbidity) and to society (economic impact) making it an important area to improve [30-32]. To assess medication compliance a self-administered questionnaire was sent to all patients twice during the Trial.

Medication compliance was measured using the Medication Adherence Reporting Scale (MARS-5). The MARS-5 is a five-item scale asking participants to indicate the frequency with which they engage in each of five components of non-compliant behaviour e.g. altering the dose or forgetting to take a dose. Since 1996, the MARS-5 has been used in studies across a variety of illnesses and in several countries [33-36] The MARS-5 has been found to have good reliability and validity [37].

Patients were also asked to comment about their beliefs and attitudes towards medicines in general and medicines prescribed for their condition. Past research has shown that levels of medication compliance are associated with patient beliefs about the necessity of taking medication [37].

#### Process of care and prescribing patterns

In order to assess the impact of PoCT on general practitioner management of the patient, the PoCT Trial measured the processes of care associated with each pathology test. The availability of a test result during the consultation should assist the general practitioner to treat and manage patients with the three conditions of interest [38]. Grieve et al [2] found that the provision of immediate test results for HbA1c led to significantly more management changes being made. The minimum number of processes of care for the Trial were based on the clinical guidelines developed for diabetes management, management of patients on anticoagulant therapy and lipids management [39-42] and depended on whether the test result was within or outside the target range. The data were collected through a case note audit (CNA) of a random sample of patient medical records. A total of 18 practices, stratified by treatment group and geographic location, were included in the sample. Sixty-five patients were then randomly selected from each of the chosen practices, or all patients were selected if the practice had less than 65 patients participating in the Trial. Following training, the auditors recorded the number and type of actions relating to each pathology test performed during the Trial. Actions included review of the test result by the general practitioner, medication review, medication changes, lifestyle advice given, referrals, blood pressure readings and requests for follow-up testing.

PoCT has the potential to improve patient compliance with medication and result in more appropriate and timely prescribing by GPs [4]. To assess the influence of PoCT on prescribing patterns, the data from the CNA were used to identify changes made to prescribed medication for each visit relating to a pathology test. These changes include dosage changes, ceasing of medication and change in type of drug. Prescribing patterns were analysed separately for test results within and outside the target range.

#### Cost effectiveness

To determine the cost effectiveness of PoCT versus pathology laboratory testing, comparative cost analysis and cost effectiveness analysis were undertaken, taking a societal perspective. Costs included in the analysis were establishment costs (equipment and training), consumable and maintenance costs, QC and QA costs, accreditation costs, costs associated with the practice consultation, testing costs, patient costs and downstream costs.

Cost data were collected from a number of sources. These included: MBS service claims (from Medicare Australia) by participating GPs; general practitioner and device operator time through a time and motion study; patient borne costs collected as part of the baseline and satisfaction questionnaires; industry sources for costs related to PoCT devices, allied health and specialist services from the Medicare Australia database; and hospitalisations from the CNA.

Cost effectiveness was measured using the incremental cost effectiveness ratio. The intermediate outcome indicator for each type of test was the proportion of patients who are maintained within the normal clinical range for that blood level based on the last test result collected during the Trial (adequate control).

#### Satisfaction

The PoCT Trial assessed the satisfaction of patients, GPs, device operators and pathology providers with PoCT, and compared this with patient, GPs and pathology provider satisfaction with usual pathology testing.

Attitudinal questions were administered at baseline (baseline questionnaires) and at the end of the Trial (satisfaction questionnaires) to patients, GPs, device operators and pathology providers. Questions covered areas such as preference, convenience, collection of blood, impact on management of conditions, impact of PoCT on the practice and difficulty in the use of PoCT devices.

Additional data relating to satisfaction were also collected through the satisfaction questionnaires. For the intervention group these covered the areas of comparative quality of the process (confidence in the collection process, confidence in the results and comparative convenience (transport, loss of work time and out of pocket expenses). The control group were asked to rate their satisfaction in the same areas as the intervention group, but as it related to pathology laboratories.

For GPs and device operators, the satisfaction questionnaire focused on their preference, attitude and stated behaviour around pathology testing. Those in the intervention group had a number of additional questions relating specifically to PoCT. Topics covered in the general practitioner and device operator questionnaires included: training; self assessed competence; accreditation method; equipment; suitability of PoCT within the consultation; perceived impact on patient health outcomes; convenience and efficiency to the practice; and payment and impact on interaction with pathology providers. Pathology provider attitudes to PoCT were also obtained, covering areas such as analytical quality, accreditation, laboratory involvement and impact on laboratory testing. For all the satisfaction questionnaires, questions were either designed specifically for the Trial or were taken from other studies [2,15,22,23,43,44].

#### Influence of geographic location

The PoCT Trial sought to determine if there were differences between urban, rural and remote geographic regions in the areas of safety, clinical effectiveness and stakeholder satisfaction.

### Data management and safety monitoring

The Data Management and Analysis Centre (DMAC) of the Discipline of Public Health at the University of Adelaide was contracted to design and implement the IT systems for the PoCT Trial. This system was comprised of a Management Information System (MIS) and a data entry system. The MIS was web-based and enabled the collection and dissemination of Trial Management information. The database into which management information was collected could also be accessed by Trial Management and Evaluation staff to produce reports as required. Access to the Trial IT systems required logins and passwords and all staff received training in its use. Data entry was performed by specialised data entry staff in DMAC following PoCT Trial standard operating procedures.

While the PoCT Trial was deemed low-risk, a Safety Subcommittee was established to monitor SAEs and incidents throughout its length, and to develop stopping rules. Practices, using a SAE Reporting Form, were required to report any SAEs for recruited patients. The SAEs were categorised as death, life threatening, permanent or significant disability or incapacity, hospitalisation, newly diagnosed cancer or other important medical event. Each SAE was initially assessed by the Trial Manager to determine the likelihood of the event resulting from involvement in the PoCT Trial before being submitted to the Safety Subcommittee for final assessment. In addition, any Trial related incident was also required to be reported to the Trial Manager for assessment using an Incident Reporting Form. Incidents could be patient, device operator, device or QC/QA related.

Throughout the PoCT Trial, all participants had access to the three organisations administering the Trial via a free-call telephone number.

### Analysis plan

#### Statistical analysis

Non-inferiority tests were planned for hypotheses relating to safety and clinical effectiveness. Comparative tests were used for hypotheses relating to cost effectiveness and satisfaction. Analyses were performed on an intention to treat basis and took into account clustering at the practice level, as well as the patient/general practitioner/device operator level where appropriate, using mixed effects models or generalised estimating equations. The level of analysis varied depending on the hypothesis (see Table 1). No adjustment was made for multiple tests of hypotheses specified a priori.

A number of potential confounders were identified a priori as important. Both unadjusted and adjusted analyses were performed. Conclusions were based on the results of the adjusted analyses.

For outcomes relating to patients or repeated measures on patients, adjustment was planned for the age and gender of the patient. For outcomes to be analysed separately for each type of test, the potential confounders to adjust for (in addition to age and gender) depended on the type of test. For tests relating to diabetes (HbA1c and microalbumin), adjustment was planned for time since diagnosis of diabetes, Aboriginal or Torres Strait Islander (ATSI), use of dietary control, prescription tablets and insulin for treating diabetes, and Body Mass Index (BMI). For HbA1c, adjustment was also planned for baseline HbA1c result. For lipids tests (total cholesterol, HDL-C and triglycerides), adjustment was planned for known heart disease, diabetes, ATSI, socio-economic status, smoking status and baseline test result. For INR, adjustment was planned for BMI, multiple co-morbidities and baseline INR result. Analyses were adjusted for all planned confounders with the exception of ATSI, due to the small number of such patients in the Trial.

The form and extent of the missing data on both outcomes and potential confounders was considered separately for each analysis. Where there was evidence to suggest the missing data were not missing completely at random, 10 completed data sets were generated for analysis using multiple imputation [45].

#### Sample size calculations

The primary outcome on which the sample size was based was the proportion of patients with test results within target range, a measure of clinical effectiveness. It was considered that PoCT would be non-inferior to pathology laboratory testing if the true difference in the proportion of patients with test results within target range (intervention minus control) was no less than -0.07. This non-inferiority margin was chosen as a compromise between clinical significance and the feasibility of recruitment.

Sample size calculations were performed separately for each type of test. Where multiple tests are performed for the same condition (e.g. HbA1c and microalbumin tests are both performed for patients with diabetes), the largest of the estimated sample sizes was used.

Based on information obtained from a pathology laboratory, the proportion of control patients with pathology results within the target range was assumed to be 0.12, 0.81, 0.77 and 0.53 for total cholesterol, HDL-C, triglycerides and HbA1c respectively. Since no suitable information was available for INR or microalbuminuria, the proportion of control patients with pathology results within the target range was assumed to be 0.5 for each of these tests to give the largest sample size. The difference in the proportion of patients with test results within target range was assumed to be zero.

A design effect of 2 was suggested in the original Trial Design to allow for correlation between observations arising from the same cluster. The Trial Evaluators investigated design effects in the Second Australian National Blood Pressure Study (ANBP2) [46] which suggested that a design effect of 2 may be conservative for a study conducted in GP. Intra-cluster correlations for the PoCT Trial will be published in a subsequent paper to assist in sample size calculations for future studies in a GP setting.

Using a one-sided, normal-approximation, non-inferiority test of two proportions, a type 1 error probability of 0.05, 80% power and assuming a design effect of 2, the number of patients required per group was 1262, 894 and 1262 for anticoagulant therapy, hyperlipidaemia and diabetes respectively.

### Ethical approval and registration

The PoCT Trial was approved by five relevant independent Australian Human Research Ethics Committees. The Trial is registered with the Australian Clinical Trial Registry, Number 12612605000272695 [47].

## Results

### Recruitment

Sixty-six practices expressed an interest in being involved in the PoCT Trial. Of these, 58 met the selection criteria and signed on to participate. Practices were then randomised resulting in 26 practices in the control group and 32 practices in the intervention group. A total of 5234 patients (2034 in the control group and 3200 in the intervention group) were recruited through these practices.

**Figure 1:**
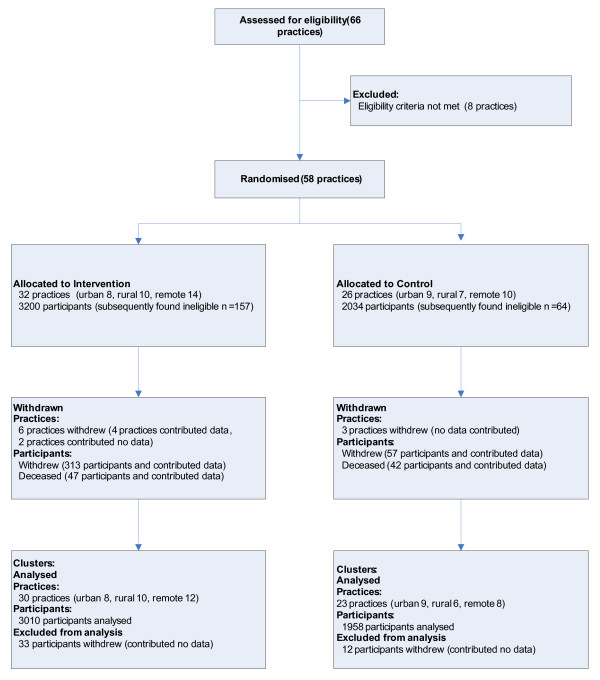
CONSORT diagram showing flow of clusters and participants' progress through the Trial.

The clusters and participants progress throughout the Trial is provided in a CONSORT diagram (Figure 1) [48].

### Patient baseline characteristics

A baseline questionnaire was sent to patients following consent and a response rate of 94.1% was achieved. Of the 4968 patients included in the analyses, 1967 had diabetes, 3819 had hyperlipidaemia and 944 were on anticoagulant therapy (patients could be registered in the Trial with one or more conditions). The characteristics of these patients were similar by treatment group and geographic location. Overall the patients tended to be older (reflecting the conditions of interest). The median age for patients on anticoagulant therapy was slightly higher than for the Trial participants overall, almost half of them reported having multiple co-morbidities and a large proportion were either overweight or obese. Patients with diabetes were primarily diet and/or tablet controlled, had been diagnosed for more than 5 years and a majority were either overweight or obese. For those with hyperlipidaemia, over a third reported having heart disease, and over a third indicated they had diabetes (see Additional file 2).

## Discussion

The PoCT Trial is a complex study taking place in a GP setting. The issues facing the implementation and design of the Trial emphasise the difficulties of undertaking a RCT in a GP setting and why RCTs in GP are often termed 'pragmatic' RCTs. As with the PoCT Trial, pragmatic RCTs are suitable to evaluate effectiveness rather than to measure efficacy [49]. Such trials determine the benefit of a treatment within routine clinical care rather than under ideal conditions. Results reflect the variation that occurs in the real world and are particularly suitable for interventions that inform policy decisions.

A key methodological issue in pragmatic RCTs is balancing internal validity and external validity. This issue was addressed for the PoCT Trial in the study design and evaluation protocol. External validity was maintained by minimising the exclusion criteria, allowing the GPs to implement PoCT in their own manner within the practice and allowing patients to choose not to have PoCT at anytime during the Trial. Internal validity was maintained through cluster randomisation of practices to reduce contamination issues.

### Evaluation protocol design

In developing the evaluation protocol and the data collection tools, three participant groups were considered – GPs and their practices, patients and pathology providers. The approach taken needed to balance data quality with the pragmatic aspects necessary when undertaking a RCT in a GP setting.

The PoCT Trial recruited a total of 58 practices located in urban, rural and remote locations across three states in Australia. The data required for the Trial needed to be collected mainly by the practices themselves so the tools had to be easy to use by GPs and device operators, not onerous to complete and be integrated into the busy fee-for-service structure of Australian GP. It was also necessary to allow for variability between the practices in terms of their size, location and capacity to participate in research and at the same time ensure quality data were collected. The practices recruited ranged from large urban based multi general practitioner practices with many practice nurses to remote solo practices with one additional staff member. The practices also varied in their capacity to undertake the Trial tasks such as recruitment of patients (for example understanding of consent, accuracy of practice databases) and understanding the importance of quality data collection. Also the original and adapted Trial Design allowed practices to implement the intervention in the ways that suited their practices. As a result, practices could either incorporate the PoCT devices into existing or new mini clinics or, more commonly, as part of the consultation process.

The PoCT Trial included patients with one or more of three conditions. This meant that any outcome measure needed to be suitable for all these conditions, rather than the most appropriate outcome for a particular condition. For example, one of the most validated methods of determining good management of anticoagulant therapy is measuring a patient's time in range. However, while this is relevant for patients on warfarin therapy, it is not a suitable measure for diabetes or lipids management. Thus, the percentage of patients and test results within target range were selected as alternative measures of clinical effectiveness as these were applicable for all three conditions. Patients also formed the largest participant group in the Trial; therefore data collection processes needed to be manageable for more than 4500 patients. Taking into account these issues, the approach taken by the PoCT Trial to collect patient data was through a series of questionnaires. This was the most efficient method of data collection and the high response rate achieved for the baseline patient questionnaire indicates that patients found this approach acceptable. Similarly the list of SAEs reported had to cover a broad range of possibilities, rather than be condition specific.

At the pathology provider level, the practices participating in the PoCT Trial utilised 23 different laboratories, representing 10 parent pathology companies, with each practice utilising on average 1.5 pathology laboratories. Pathology results were required for several of the hypotheses. Each pathology company used different testing reagents and different electronic record systems and considerable work was required to capture these data in a format that could be used for the Trial.

The approach taken by the Trial Evaluation group was to minimise the data collection undertaken by the GPs and practices and where it was necessary for them to collect data, incorporate this as much as possible into their everyday practice. An example was the development of the PoCT request/result form for the intervention practices. This provided the practice with documentation of the test request and result using the PoCT devices, but the duplicate form was used by the Trial as a record of test results. This form was also provided to the practices in an electronic format that could be included in their electronic medical record systems.

### Recruitment

The PoCT Trial retained 84.5% of practices and 86.1% of patients. These high retention rates are likely to reflect the interest by GPs and patients in PoCT and the effort made by the researchers to minimise the impact on both the practices and patients. Various support strategies known to improve recruitment and compliance [50] were implemented, including NSOs, a telephone support line and regular newsletters, all of which may have contributed to the continuation of practices in the Trial. At the same time, the intervention practices reported that patients had a high level of interest in PoCT. Through the use of the data collection questionnaires, patients were contacted regularly by the researchers during the Trial.

### Limitations

Unfortunately, the PoCT Trial was not able to recruit sufficient patients in two of the three conditions to obtain desired power. The Trial Design required the recruitment of practices in three geographic locations – urban, rural and remote. However, the original Trial Design did not consider the possibility that practices in rural or remote locations would not have sufficient patient population to meet the required sample size and hence the adapted Trial Design allowed practices to be recruited knowing that they could not meet the minimum requirement of patients.

With any trial, ensuring adherence to the evaluation and treatment protocol by participants is difficult, although this is of less importance in a pragmatic RCT [49]. This difficulty was amplified for the PoCT Trial because of the large number of participants and their geographic spread. One of the aims of establishing NSOs in each region was to provide a local link for practices and for the NSO to monitor adherence to the protocol, which worked to some extent. However, it was reliant on the relationship between the NSO and the practices and the geographic spread of practices meant that it was not possible for the NSO to visit practices on a regular basis.

To maximise time the CNA of patient records was undertaken for only a sample of patients. The descriptive analysis suggests that the sample of patients was representative of the entire patient population of the PoCT Trial in terms of baseline characteristics and hence, results based on analysis of CNA data are applicable to all Trial patients.

## Conclusion

The PoCT Trial is one of the largest and most comprehensive RCTs to evaluate the impact of PoCT in a GP setting. There are few RCTs in this area and none have investigated all the areas covered in this Trial or at the scale of this Trial either in terms of the number of practices, the number of patients or the number of pathology tests included [8,18,27,28,51-53]. No previous trials and very few observational studies [16,17] have investigated the influence of geographic location on PoCT. The results of the PoCT Trial should provide a sound evidence base as to whether PoCT (for the three conditions) should be implemented by the government in Australian general practice.

## Competing interests

The authors declare that they have no competing interests.

## Authors' contributions

CL and JB obtained funding for the Trial Management and Evaluation. CL, AG, TB and BG drafted the Trial Management and Evaluation protocols. LY, PR and KW drafted the statistical analysis plan. JB, PR, LY, KW, JG and MS critically reviewed and revised the evaluation protocol. JG obtained funding and designed the protocol for the provision of QA testing. MS obtained funding and designed the protocol for the provision of QC testing. All authors read and approved the final manuscript.

## Supplementary Material

Additional File 1 Schedule of data collection activities throughout the PoCT Trial. Click here for file

Additional File 2 Comparison of patient baseline characteristics by condition. Click here for file
