# A pragmatic cluster randomised controlled trial to evaluate the safety, clinical effectiveness, cost effectiveness and satisfaction with point of care testing in a general practice setting – rationale, design and baseline characteristics

**DOI:** 10.1186/1745-6215-10-7

**Published:** 2009-01-28

**Authors:** Caroline Laurence, Angela Gialamas, Lisa Yelland, Tanya Bubner, Philip Ryan, Kristyn Willson, Briony Glastonbury, Janice Gill, Mark Shephard, Justin Beilby, for members of the PoCT Trial Management Committee

**Affiliations:** 1 Discipline of General Practice, The University of Adelaide, Adelaide, South Australia, Australia; 2 Discipline of Public Health, The University of Adelaide, Adelaide, South Australia, Australia; 3 Save Sight Institute, University of Sydney, Sydney, New South Wales, Australia; 4 RCPA Quality Assurance Programs Pty Ltd, Adelaide, South Australia, Australia; 5 Community Point-of-Care Services, Flinders University Rural Clinical School, Flinders University, Adelaide, South Australia, Australia; 6 Faculty of Health Sciences, The University of Adelaide, Adelaide, South Australia, Australia

Following the publication of our article [1] we noticed an error within Additional file 2: Comparison of patient baseline characteristics by condition. The column headings 'Control' and 'Intervention' are incorrectly positioned in the table and should be switched.

The table should therefore appear as shown in Table 1 in this correction.

**Table 1:** Comparison of patient baseline characteristics by condition

Condition Group	Characteristic		Control n (%)	Intervention n (%)	Total n (%)
Diabetes			N = 785	N = 1182	N = 1967
	Male		432 (55.0)	651 (55.1)	1083 (55.1)
	Age (years)	Median (IQ range)	67.0 (59.0–74.0)	66.0 (58.0–73.0)	66.0 (59.0–73.0)
	Aboriginal and Torres Strait Islander	Yes	4 (0.5)	23 (1.9)	27 (1.4)
		No	723 (92.1)	1036 (87.6)	1759 (89.4)
		Missing	58 (7.4)	123 (10.4)	181 (9.2)
	BMI	Underweight	5 (0.6)	3 (0.3)	8 (0.4)
		Normal	121 (15.4)	181 (15.3)	302 (15.4)
		Overweight	243 (31.0)	386 (32.7)	629 (32.0)
		Obese	334 (42.5)	464 (39.3)	798 (40.6)
		Missing	82 (10.4)	148 (12.5)	230 (11.7)
	HbA1c %	Median (IQ range)	7.1 (6.3–8.0)	6.9 (6.2–7.7)	7.0 (6.3–7.8)
		Missing	64 (8.2)	90 (7.6)	154 (7.8)
	Diabetes management:				
	Dietary control	Yes	446 (56.8)	604 (51.1)	1050 (53.4)
		No	309 (39.4)	503 (42.6)	812 (41.3)
		Missing	30 (3.8)	75 (6.3)	105 (5.3)
	Insulin	Yes	145 (18.5)	204 (17.3)	349 (17.7)
		No	610 (77.7)	903 (76.4)	1513 (76.9)
		Missing	30 (3.8)	75 (6.3)	105 (5.3)
	Prescription tablets	Yes	541 (68.9)	729 (61.7)	1270 (64.6)
		No	214 (27.3)	378 (32.0)	592 (30.1)
		Missing	30 (3.8)	75 (6.3)	105 (5.3)
	Years since first diagnosed	< 1 year	39 (5.0)	67 (5.7)	106 (5.4)
		1–5 years	256 (32.6)	403 (34.1)	659 (33.5)
		6–10 years	201 (25.6)	235 (19.9)	436 (22.2)
		> 10 years	230 (29.3)	311 (26.3)	541 (27.5)
		Missing	59 (7.5)	166 (14.0)	225 (11.4)
Hyperlipidaemia			N = 1463	N = 2356	N = 3819
	Male		753 (51.5)	1277 (54.2)	2030 (53.2)
	Age (years)	Median (IQ range)	68.0 (60.0–74.0)	66.0 (58.0–74.0)	66.0 (59.0–74.0)
	Aboriginal and Torres Strait Islander	Yes	9 (0.6)	26 (1.1)	35 (0.9)
		No	1342 (91.7)	2102 (89.2)	3444 (90.2)
		Missing	112 (7.7)	228 (9.7)	340 (8.9)
	Index of Relative Socio-Economic Disadvantage	1^st ^quartile	251 (17.2)	575 (24.4)	826 (21.6)
		2^nd ^quartile	476 (32.5)	756 (32.1)	1232 (32.3)
		3^rd ^quartile	390 (26.7)	663 (28.1)	1053 (27.6)
		4^th ^quartile	346 (23.7)	362 (15.4)	708 (18.5)
	Co-morbid conditions:				
	Known heart disease	Yes	540 (36.9)	812 (34.5)	1352 (35.4)
		No	869 (59.4)	1398 (59.3)	2267 (59.4)
		Missing	54 (3.7)	146 (6.2)	200 (5.2)
	Diabetes		509 (34.8)	853 (36.2)	1362 (35.7)
	Smoking status	Current	103 (7.0)	201 (8.5)	304 (8.0)
		Ex-smoker	644 (44.0)	1001 (42.5)	1645 (43.1)
		Never smoked	654 (44.7)	990 (42.0)	1644 (43.0)
		Missing	62 (4.2)	164 (7.0)	226 (5.9)
	Total cholesterol	Median (IQ range)	4.7 (4.0–5.4)	4.6 (4.0–5.4)	4.7 (4.0–5.4)
		Missing	99 (6.8)	186 (7.9)	285 (7.5)
	Triglyceride	Median (IQ range)	1.6 (1.1–2.2)	1.6 (1.2–2.2)	1.6 (1.2–2.2)
		Missing	185 (12.6)	204 (8.7)	389 (10.2)
	HDL-C	Median (IQ range)	1.3 (1.1–1.6)	1.3 (1.1–1.5)	1.3 (1.1–1.6)
		Missing	239 (16.3)	515 (21.9)	754 (19.7)
Anticoagulant therapy			N = 372	N = 572	N = 944
	Male		217 (58.3)	326 (57.0)	543 (57.5)
	Age (years)	Median (IQ range)	73.0 (66.0–79.0)	73.0 (65.0–78.0)	73.0 (65.0–79.0)
	BMI	Underweight	7 (1.9)	5 (0.9)	12 (1.3)
		Normal	90 (24.2)	165 (28.8)	255 (27.0)
		Overweight	130 (34.9)	190 (33.2)	320 (33.9)
		Obese	111 (29.8)	132 (23.1)	243 (25.7)
		Missing	34 (9.1)	80 (14.0)	114 (12.1)
	Multiple co-morbidities	Yes	170 (45.7)	285 (49.8)	455 (48.2)
		No	186 (50.0)	259 (45.3)	445 (47.1)
		Missing	16 (4.3)	28 (4.9)	44 (4.7)
	INR	Below target range	73 (19.6)	115 (20.1)	188 (19.9)
		Within target range	197 (53.0)	316 (55.2)	513 (54.3)
		Above target range	37 (9.9)	63 (11.0)	100 (10.6)
		Missing	65 (17.5)	78 (13.6)	143 (15.1)
