# Supplementary material for: A pragmatic cluster randomised controlled trial to evaluate the safety, clinical effectiveness, cost effectiveness and satisfaction with point of care testing in a general practice setting – rationale, design and baseline characteristics
Source: Trials. 2008 Aug 6;9:50. doi: 10.1186/1745-6215-9-50 (PMC2519057; doi:10.1186/1745-6215-9-50)
Supplement: Additional File 1 — Schedule of data collection activities throughout the PoCT Trial. [file 1745-6215-9-50-S1.doc]

Schedule of data collection activities

| **Measure** | **Participant Group** | **Method** | **Baseline** | **Continuous** | **6 months** | **14 months** | **18 months** |
| --- | --- | --- | --- | --- | --- | --- | --- |
| Background data | Patient | Questionnaire | X |  |  |  |  |
|  | Practice | Questionnaire | X |  |  |  |  |
|  | General Practitioner | Questionnaire | X |  |  |  |  |
|  | Device Operator | Questionnaire | X |  |  |  |  |
|  | Pathology Provider | Questionnaire | X |  |  |  |  |
| Attitudes to pathology testing | Patient | Questionnaire | X |  |  |  | X |
|  | General Practitioner | Questionnaire | X |  |  |  | X |
|  | Device Operator | Questionnaire | X |  |  |  | X |
| Comparison of laboratory and PoCT results | Practice | Pathology results  PoCT results |  |  | X |  |  |
| Practice costing data | Practice | Medicare Australia |  |  |  |  | X |
|  |  | Time and motion study |  |  |  | X |  |
| Patient Costing data | Patient | Questionnaire |  |  |  |  | X |
| Medication compliance and lifestyle advice | Patient | Questionnaire |  |  | X | X |  |
| Incidents |  | Reporting Form |  | X |  |  |  |
| Serious adverse events |  | Reporting Form |  | X |  |  |  |
| Process of care actions | General Practitioner | Case note audit |  |  |  |  | X |
| Appropriate prescribing | Patient/Practice/ General Practitioner | Case note audit/ Medicare Australia |  |  |  |  | X |
| Improved therapeutic control | Patient | PoCT/Pathology results | X | X |  |  |  |
| Satisfaction survey | Patient | Questionnaire |  |  |  |  | X |
|  | General Practitioner | Questionnaire |  |  |  |  | X |
|  | Device Operator | Questionnaire |  |  |  |  | X |
|  | Pathology Provider | Questionnaire |  |  |  |  | X |
