# Supplementary material for: A pragmatic cluster randomised controlled trial to evaluate the safety, clinical effectiveness, cost effectiveness and satisfaction with point of care testing in a general practice setting – rationale, design and baseline characteristics
Source: Trials. 2008 Aug 6;9:50. doi: 10.1186/1745-6215-9-50 (PMC2519057; doi:10.1186/1745-6215-9-50)
Supplement: Additional File 2 — Comparison of patient baseline characteristics by condition. [file 1745-6215-9-50-S2.doc]

Comparison of patient baseline characteristics by condition

| **Condition Group** | **Characteristic** |  | **Intervention n (%)** | **Control n (%)** | **Total n (%)** |
| --- | --- | --- | --- | --- | --- |
| **Diabetes** |  |  | N=785 | N=1182 | N=1967 |
|  | Male |  | 432 (55.0) | 651 (55.1) | 1083 (55.1) |
|  | Age (years) | Median (IQ range) | 67.0 (59.0-74.0) | 66.0 (58.0-73.0) | 66.0 (59.0-73.0) |
|  | Aboriginal and Torres Strait Islander | Yes | 4 (0.5) | 23 (1.9) | 27 (1.4) |
|  |  | No | 723 (92.1) | 1036 (87.6) | 1759 (89.4) |
|  |  | Missing | 58 (7.4) | 123 (10.4) | 181 (9.2) |
|  | BMI | Underweight | 5 (0.6) | 3 (0.3) | 8 (0.4) |
|  |  | Normal | 121 (15.4) | 181 (15.3) | 302 (15.4) |
|  |  | Overweight | 243 (31.0) | 386 (32.7) | 629 (32.0) |
|  |  | Obese | 334 (42.5) | 464 (39.3) | 798 (40.6) |
|  |  | Missing | 82 (10.4) | 148 (12.5) | 230 (11.7) |
|  | HbA1c % | Median (IQ range) | 7.1 (6.3-8.0) | 6.9 (6.2-7.7) | 7.0 (6.3-7.8) |
|  |  | Missing | 64 (8.2) | 90 (7.6) | 154 (7.8) |
|  | Diabetes management: |  |  |  |  |
|  | Dietary control | Yes | 446 (56.8) | 604 (51.1) | 1050 (53.4) |
|  |  | No | 309 (39.4) | 503 (42.6) | 812 (41.3) |
|  |  | Missing | 30 (3.8) | 75 (6.3) | 105 (5.3) |
|  | Insulin | Yes | 145 (18.5) | 204 (17.3) | 349 (17.7) |
|  |  | No | 610 (77.7) | 903 (76.4) | 1513 (76.9) |
|  |  | Missing | 30 (3.8) | 75 (6.3) | 105 (5.3) |
|  | Prescription tablets | Yes | 541 (68.9) | 729 (61.7) | 1270 (64.6) |
|  |  | No | 214 (27.3) | 378 (32.0) | 592 (30.1) |
|  |  | Missing | 30 (3.8) | 75 (6.3) | 105 (5.3) |
|  | Years since first diagnosed | < 1 year | 39 (5.0) | 67 (5.7) | 106 (5.4) |
|  |  | 1-5 years | 256 (32.6) | 403 (34.1) | 659 (33.5) |
|  |  | 6-10 years | 201 (25.6) | 235 (19.9) | 436 (22.2) |
|  |  | > 10 years | 230 (29.3) | 311 (26.3) | 541 (27.5) |
|  |  | Missing | 59 (7.5) | 166 (14.0) | 225 (11.4) |
| **Hyperlipidaemia** |  |  | N=1463 | N=2356 | N=3819 |
|  | Male |  | 753 (51.5) | 1277 (54.2) | 2030 (53.2) |
|  | Age (years) | Median (IQ range) | 68.0 (60.0-74.0) | 66.0 (58.0-74.0) | 66.0 (59.0-74.0) |
|  | Aboriginal and Torres Strait Islander | Yes | 9 (0.6) | 26 (1.1) | 35 (0.9) |
|  |  | No | 1342 (91.7) | 2102 (89.2) | 3444 (90.2) |
|  |  | Missing | 112 (7.7) | 228 (9.7) | 340 (8.9) |
|  | Index of Relative Socio-Economic Disadvantage | 1^st^ quartile | 251 (17.2) | 575 (24.4) | 826 (21.6) |
|  |  | 2^nd^ quartile | 476 (32.5) | 756 (32.1) | 1232 (32.3) |
|  |  | 3^rd^ quartile | 390 (26.7) | 663 (28.1) | 1053 (27.6) |
|  |  | 4^th^ quartile | 346 (23.7) | 362 (15.4) | 708 (18.5) |
|  | Co-morbid conditions: |  |  |  |  |
|  | Known heart disease | Yes | 540 (36.9) | 812 (34.5) | 1352 (35.4) |
|  |  | No | 869 (59.4) | 1398 (59.3) | 2267 (59.4) |
|  |  | Missing | 54 (3.7) | 146 (6.2) | 200 (5.2) |
|  | Diabetes |  | 509 (34.8) | 853 (36.2) | 1362 (35.7) |
|  | Smoking status | Current | 103 (7.0) | 201 (8.5) | 304 (8.0) |
|  |  | Ex-smoker | 644 (44.0) | 1001 (42.5) | 1645 (43.1) |
|  |  | Never smoked | 654 (44.7) | 990 (42.0) | 1644 (43.0) |
|  |  | Missing | 62 (4.2) | 164 (7.0) | 226 (5.9) |
|  | Total cholesterol | Median (IQ range) | 4.7 (4.0-5.4) | 4.6 (4.0-5.4) | 4.7 (4.0-5.4) |
|  |  | Missing | 99 (6.8) | 186 (7.9) | 285 (7.5) |
|  | Triglyceride | Median (IQ range) | 1.6 (1.1-2.2) | 1.6 (1.2-2.2) | 1.6 (1.2-2.2) |
|  |  | Missing | 185 (12.6) | 204 (8.7) | 389 (10.2) |
|  | HDL-C | Median (IQ range) | 1.3 (1.1-1.6) | 1.3 (1.1-1.5) | 1.3 (1.1-1.6) |
|  |  | Missing | 239 (16.3) | 515 (21.9) | 754 (19.7) |
| **Anticoagulant therapy** | |  | N=372 | N=572 | N=944 |
|  | Male |  | 217 (58.3) | 326 (57.0) | 543 (57.5) |
|  | Age (years) | Median (IQ range) | 73.0 (66.0-79.0) | 73.0 (65.0-78.0) | 73.0 (65.0-79.0) |
|  | BMI | Underweight | 7 (1.9) | 5 (0.9) | 12 (1.3) |
|  |  | Normal | 90 (24.2) | 165 (28.8) | 255 (27.0) |
|  |  | Overweight | 130 (34.9) | 190 (33.2) | 320 (33.9) |
|  |  | Obese | 111 (29.8) | 132 (23.1) | 243 (25.7) |
|  |  | Missing | 34 (9.1) | 80 (14.0) | 114 (12.1) |
|  | Multiple co-morbidities | Yes | 170 (45.7) | 285 (49.8) | 455 (48.2) |
|  |  | No | 186 (50.0) | 259 (45.3) | 445 (47.1) |
|  |  | Missing | 16 (4.3) | 28 (4.9) | 44 (4.7) |
|  | INR | Below target range | 73 (19.6) | 115 (20.1) | 188 (19.9) |
|  |  | Within target range | 197 (53.0) | 316 (55.2) | 513 (54.3) |
|  |  | Above target range | 37 (9.9) | 63 (11.0) | 100 (10.6) |
|  |  | Missing | 65 (17.5) | 78 (13.6) | 143 (15.1) |
